# Intramolecular Charge‐Transfer Dopants Enable Isolated Triplet Excitons as Spin Qutrits in a Single Crystal

**DOI:** 10.1002/anie.202524670

**Published:** 2026-03-09

**Authors:** Yaoyao Han, Samuel B. Tyndall, Kathryn R. Peinkofer, Yuheng Huang, Ryan M. Young, Matthew D. Krzyaniak, Michael R. Wasielewski

**Affiliations:** ^1^ Department of Chemistry Institute for Quantum Information Research and Engineering and Center for Molecular and Quantum Transduction Northwestern University Evanston Illinois USA

**Keywords:** doped organic single crystal, EPR spectroscopy, excitonic spin, time‐resolved spectroscopy

## Abstract

Organic molecular crystals enable spin alignment of triplet excitons over macroscopic distances, offering a molecular route to solid‐state quantum technologies. In typical crystals, however, dense packing promotes spin decoherence through dipolar coupling and exciton hopping. Although dilution doping can mitigate these effects, reported examples remain scarce because dopants must satisfy stringent structural and energetic constraints. Here, we broaden the design space for doped organic crystals by introducing a strategy that employs a host‐derived dopant with intramolecular charge‐transfer (ICT) character that maintains structural compatibility with the host lattice, while its ICT character lowers the triplet energy to localize triplet excitons. Ultrafast transient absorption spectroscopy confirms triplet formation, while time‐resolved electron paramagnetic resonance (TREPR) spectroscopy reveals that these oriented triplets possess selectively addressable spin sublevel transitions. Additionally, pulse‐EPR measurements yield a phase memory time (*T*_m_) of 7.1 µs at 10 K and 3.5 µs at 85 K, enabled by reduced electron‐electron dipolar coupling and suppressed exciton hopping. Temperature‐dependent studies further show that coherence is ultimately limited by nuclear spin flip–flops at low temperatures and spin‐phonon coupling at higher temperatures. These findings demonstrate that ordered triplet excitons produced by an ICT dopant in single crystals promote spin coherence at elevated temperatures.

## Graphical abstract

Photoexcitation of an intramolecular charge‐transfer molecule doped into single crystals of a structurally similar host molecule results in well‐ordered, spectrally addressable, and orientation‐tunable triplet excitons that maintain coherence at elevated temperatures.

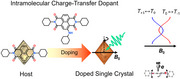

## 1 Introduction

The development of robust solid‐state qubits hold transformative potential for quantum information science [1, 2, 3, 4, 5, 6, 7]. Tremendous progress has been achieved across diverse solid‐state platforms. Superconducting circuits [4] and semiconductor quantum dots [3] have demonstrated remarkable quantum control, although their operation generally requires ultralow temperatures. Solid‐state defect systems, exemplified by nitrogen‐vacancy (NV^−^) centers in diamond have extended coherent spin operation to ambient conditions and established one of the most mature qubit architectures for room‐temperature quantum sensing [7]. However, achieving atomically precise and scalable fabrication of defect qubits remains a technical challenge.

In this context, molecular systems that generate paramagnetic excitons offer a promising alternative [5, 6, 8, 9]. Their large excited‐state energy gaps, combined with spin‐selective relaxation pathways, enable optical initialization of well‐defined multilevel quantum states (*S* ≥ 1) even at room temperature [10, 11, 12, 13, 14, 15]. Moreover, chemical synthesis allows for atomic‐level control over the optical and spin properties while offering a promising route toward scalable molecular quantum materials [5, 6, 8]. Engineering organic crystals further extends these advantages by leveraging the intrinsic crystallinity of molecular assemblies to construct ordered spin arrays. In particular, the three spin sublevels (*T*_+_, *T*_0_, and *T*_−_) of photogenerated triplet excitons (*S* = 1) can serve as *qutrits* because ordering them in single crystals results in narrow resonances, whose frequency spacing can be adjusted by rotating the crystal relative to an externally applied magnetic field [16, 17], which results in spectrally addressable, well‐defined spin transitions [16, 17, 18, 19, 20, 21]. Such control can bring the frequencies of the transitions between the triplet spin sublevels within typical microwave resonator bandwidths, enabling their selective or simultaneous manipulation, which is essential for implementing high‐order quantum gates in multilevel spin systems [22].

However, maintaining long triplet spin coherence in molecular crystals required for qutrit operations remains challenging as dense molecular packing enhances multiple decoherence pathways. Among these, electron–electron dipolar interactions [16, 17, 19, 23] and exciton hopping [17, 18, 19, 24, 25] are particularly pronounced. Decoherence from electron–electron dipolar interactions originate from stochastic fluctuations of the local dipolar fields, which are generated by random spin flips of neighboring spins driven either by spin–lattice relaxation or by spin diffusion through flip–flop processes [23, 26]. This effect is therefore sensitive to the number of spins and becomes more pronounced at higher spin densities [23, 26]. In crystals with magnetically inequivalent sites, exciton hopping stochastically modulates the effective spin Hamiltonian, producing spectral diffusion and consequently accelerating ensemble dephasing on the time scale of the exciton hopping [25].

In single‐component crystals and cocrystals, decoherence arising from electron–electron dipolar interactions can be partially mitigated by reducing the density of spins in the crystal (e.g., via weak excitation) [17, 19]. Nevertheless, decoherence associated with exciton hopping remains difficult to suppress owing to its low activation barrier (∼20 meV) [17, 18, 19]. As a result, although photogenerated triplet excitons in these crystals can be initialized via intersystem crossing (ISC) or singlet fission (SF) at elevated temperatures, long spin coherence times are typically sustained only at much lower temperatures (<60 K). [17, 24, 27] To mitigate exciton‐hopping‐induced decoherence, several crystal‐engineering strategies have been developed [16, 19, 28]. Engineering cocrystals with π‐stacked molecules occupying magnetically equivalent sites can reduce spectral diffusion, as exciton hopping between such sites maintains the spin environment [16]. Alternatively, functionalizing chromophores with bulky substituents [19] or immobilizing them in nanoporous crystalline materials [14] affords control over intermolecular coupling that limits triplet diffusion.

A more direct approach to suppressing exciton hopping is to employ doped single crystals, in which a low concentration of spins is confined within inert host lattices, thereby increasing the energetic barrier for diffusion [13, 28, 29]. However, this strategy is constrained by rigorous structural and energetic requirements: the dopant must have a lower excited‐state energy than the host to enable selective excitation and exciton localization, while maintaining a nearly identical molecular shape and size to preserve lattice compatibility. As a result, it has been realized in only a few purely organic systems, predominantly based on linear aromatic hydrocarbons such as tetracene [29, 30] or pentacene [13, 28, 30, 31, 32]. Among these, pentacene‐doped *p*‐terphenyl crystals stand out as one of the most mature doped crystal systems, as they sustain triplet exciton spin coherence even at room temperature, demonstrating the significant promise of doped molecular crystals for practical quantum technologies. Hence, developing new doping strategies offers a promising avenue to address the limited number of doped crystal systems and expand the library of doped molecular crystals, thereby further advancing their potential for molecular quantum technologies.

**FIGURE 1:**
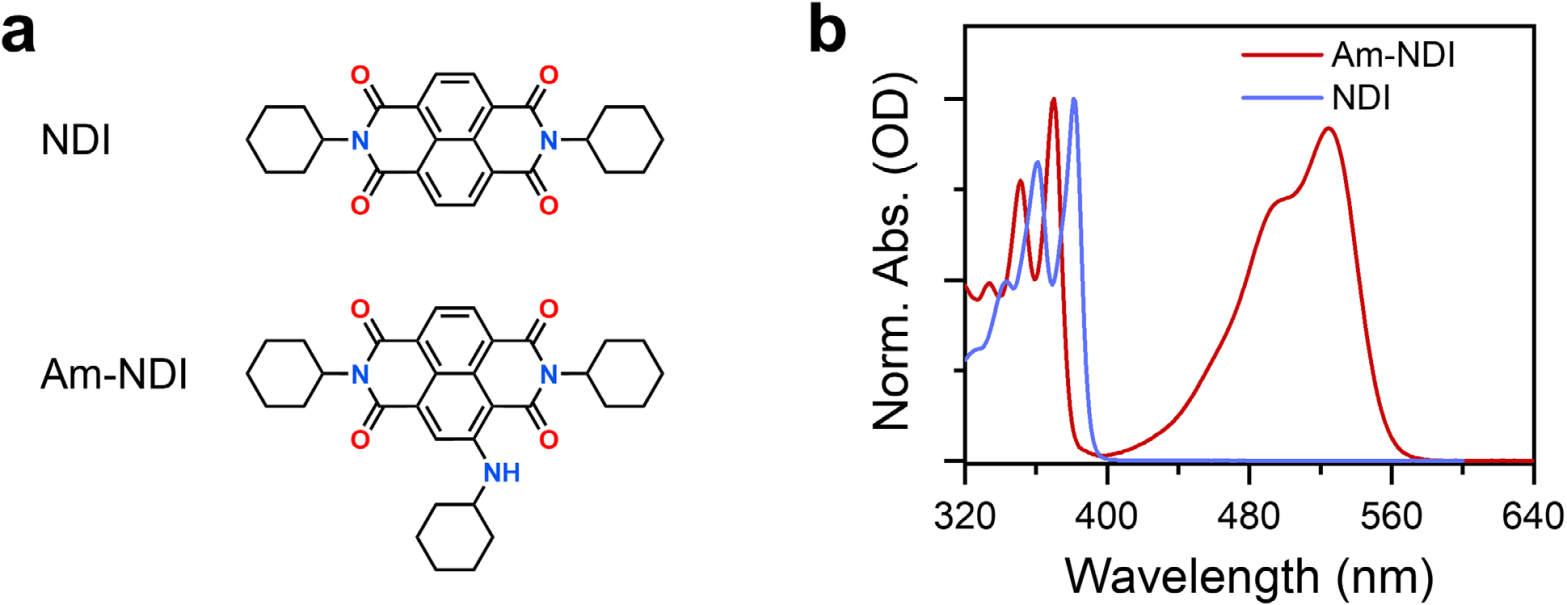
(a) Structures of NDI and Am‐NDI. (b) UV–visible spectra of NDI and Am‐NDI in CH_2_Cl_2_ at 295 K.

In this work, we designed a new doped organic crystal by using a host‐derived dopant that exhibits intramolecular charge‐transfer (ICT) interactions, which enables the photogenerated triplet state of the dopant to display enhanced spin coherence properties. The molecular structures of the host and dopant are shown in Figure 1a. The host material, *N,N′‐*dicyclohexyl‐naphthalene‐1,4:5,8‐bis(dicarboximide) (NDI) can be grown into millimeter‐sized, high‐quality single crystals exhibiting well‐ordered molecular packing [33]. The dopant, 2‐(cyclohexylamino)‐*N,N′‐*dicyclohexyl‐naphthalene‐1,4:5,8‐bis(dicarboximide) (Am‐NDI), retains the host‐like molecular framework, which enables its seamless incorporation into the NDI lattice without significantly disrupting the crystal packing. In addition, the amino substituent introduces intrinsic ICT character, thereby lowering both singlet and triplet energies. Upon photoexcitation, Am‐NDI undergoes intersystem crossing (ISC) to generate localized triplet excitons. The spatial alignment of these excitons within the doped crystal lattice enables tunable and individually addressable spin sublevel transitions needed for qutrit function. Pulse‐EPR measurements show that the spin phase memory time (*T*_m_), which is closely related to the spin coherence time (*T*_2_) [34] is 7.1 µs at 10 K and 3.5 µs at 85 K. This long coherence time arises from dilution doping, which reduces dipolar coupling, and a 190 meV dopant–host triplet energy offset that suppresses thermally activated triplet hopping. Thus, *T*_m_ is limited by nuclear spin flip–flops at low temperatures and spin–phonon coupling at elevated temperatures. These findings demonstrate the effectiveness of host‐derived ICT doping for realizing spatially isolated triplet excitons with extended spin coherence lifetimes in organic molecular crystals.

## 2 Results and Discussion

### 2.1 Solution‐State Optical Properties

Synthetic procedures for the host NDI and the dopant Am‐NDI (Figure 1a) are described in the Supporting Information. The UV–visible absorption spectra of the NDI and Am‐NDI in CH_2_Cl_2_ are shown in Figure 1b. As expected, NDI exhibits a structured absorption band between 320 and 400 nm, characteristic of its ^1^(π–π*) transition [35]. With an amino substituent, Am‐NDI displays two distinct absorption features. The higher‐energy band retains the vibronic progression observed in NDI while being slightly blue‐shifted by ∼10 nm. In addition, a pronounced lower‐energy band appears at 525 nm, which is assigned to an intramolecular charge‐transfer (ICT) transition from the electron‐donating amino substituent to the electron‐deficient NDI core. Comparable ICT bands have been reported for NDI derivatives functionalized with donor substituents [36, 37, 38], further supporting this assignment.

The ICT interaction induces a significant singlet‐energy offset (∼0.9 eV) between NDI and Am‐NDI, providing a distinct spectral separation that permits selective excitation of the dopant. Moreover, the triplet energy of Am‐NDI is lowered by 190 meV, as revealed by phosphorescence spectroscopy (Figure S2), thereby promoting efficient triplet exciton trapping at the dopant site and fulfilling a key requirement for triplet localization in doped molecular crystals.

**FIGURE 2:**
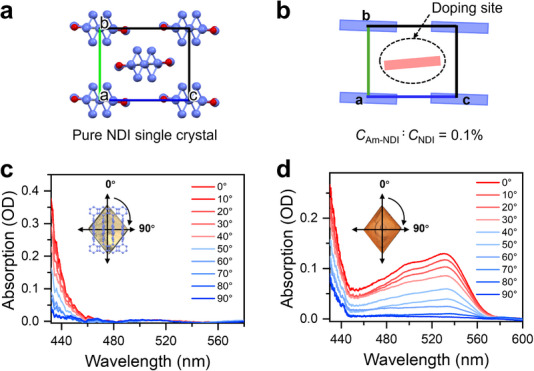
(a) Crystal structure of NDI viewed along the *a*‐axis. Carbon, nitrogen, and oxygen atoms are shown in blue, dark gray, and red, respectively. Hydrogen atoms are omitted for clarity. (b) Schematic illustration of Am‐NDI dopant incorporation into the NDI crystal lattice. NDI molecules and Am‐NDI are highlighted in blue and pink, respectively. Polarization‐resolved steady‐state absorption spectra of (c) an NDI single crystal and (d) Am‐NDI‐doped NDI single crystal. The angles indicate the orientation of the linearly polarized probe light relative to the crystallographic *a‐*axis and the molecular packing, as illustrated in the insets.

***Crystal Structure***. Crystals of NDI were grown using vapor diffusion methods, as detailed in the Supporting Information, affording large pale‐yellow single crystals with block‐like morphology and slightly skewed parallelogram facets (Figure 2c, inset). The unit cell structure is shown in Figure 2a, and additional crystallographic data obtained from single‐crystal x‐ray diffraction are summarized in Table S1. NDI crystallizes in the P2_1_/c space group (*a* = 18.30 Å, *b* = 6.63 Å, and *c* = 8.41 Å), consistent with a previous report [33], with the asymmetric unit containing a single unique molecule. Within the crystal lattice, the NDIs exhibit slip‐stacked π–π interactions along the *b*‐axis, forming a brickwork packing motif. A pronounced lateral displacement (∼4.20 Å) along the *c*‐axis between adjacent NDI cores is likely driven by steric repulsion from the cyclohexyl groups [33]. This arrangement confines each NDI molecule within a box‐like environment, providing sufficient space to accommodate Am‐NDI dopants. Notably, the molecular *x*‐axes of all NDIs align with the crystallographic *a‐*axis, which corresponds to the macroscopic long axis of the crystal (Figure S4). Their *z*‐axes are tilted by ∼5° relative to one another (Figure S3). Nevertheless, the overall molecular orientation remains highly ordered. This high degree of structural order is also manifested in the polarization‐resolved steady‐state absorption spectra. As shown in Figure 2c, NDI single crystals exhibit absorption below 460 nm, red‐shifted relative to their solution spectra due to the dielectric environment and molecular packing. Limited by our instrument detection, the main short‐wavelength absorption peak is not resolved and only its tail is visible in the spectra. The spectra show maximum intensity when the incident polarization is aligned with the crystallographic *a‐*axis (i.e., the molecular *x*‐axis) and reaches a minimum at 90°, where the polarization is along the molecular *z*‐axis. This pronounced anisotropy reflects the orientation of the NDI π–π* transition dipole, which lies along the molecular *x*‐axis with negligible contribution along the *z*‐axis.

Crystals doped with Am‐NDI were grown by the same method as that used for pure NDI (molar doping concentration: 0.1%, see Supporting Information for details). The doped crystals display the same block‐like morphology (Figure 2d, inset). Importantly, the doped crystals exhibit a pronounced orange color compared to the pale yellow of pure NDI crystals, indicating the incorporation of Am‐NDI. Within the resolution of x‐ray diffraction data, no structural differences can be discerned between doped and pure NDI crystals (Tables S1 and S2), suggesting that the host lattice remains essentially unaltered on the average structural level. However, the orientation of the dopant molecules remains unresolved by this technique.

Polarization‐resolved absorption spectra, being highly sensitive to molecular alignment, were employed to verify the dopant orientation. As illustrated in Figure 2d, absorption below 450 nm is attributed to the host NDI, while the 450–580 nm region corresponds to Am‐NDI. Both bands exhibit the same polarization dependence, with maximum intensity along the crystallographic *a‐*axis, confirming that dopant and host molecules adopt the same orientation within the lattice (Figure 2b). This orientation assignment is also corroborated by single‐crystal TREPR measurements discussed below. Additionally, polarization‐dependent spectra recorded from 0° to 90° show a pronounced decrease in intensity but essentially identical band shapes, indicating the absence of intermolecular CT states, which is consistent with their weak π–π interactions caused by the slip‐stacked arrangement [39] and the minimal energetic propensity for intermolecular charge transfer [40]. The Am‐NDI absorption band also retains resolved vibronic features, pointing to a homogeneous dopant distribution.

### 2.2 Transient Absorption Spectroscopy

**FIGURE 3:**
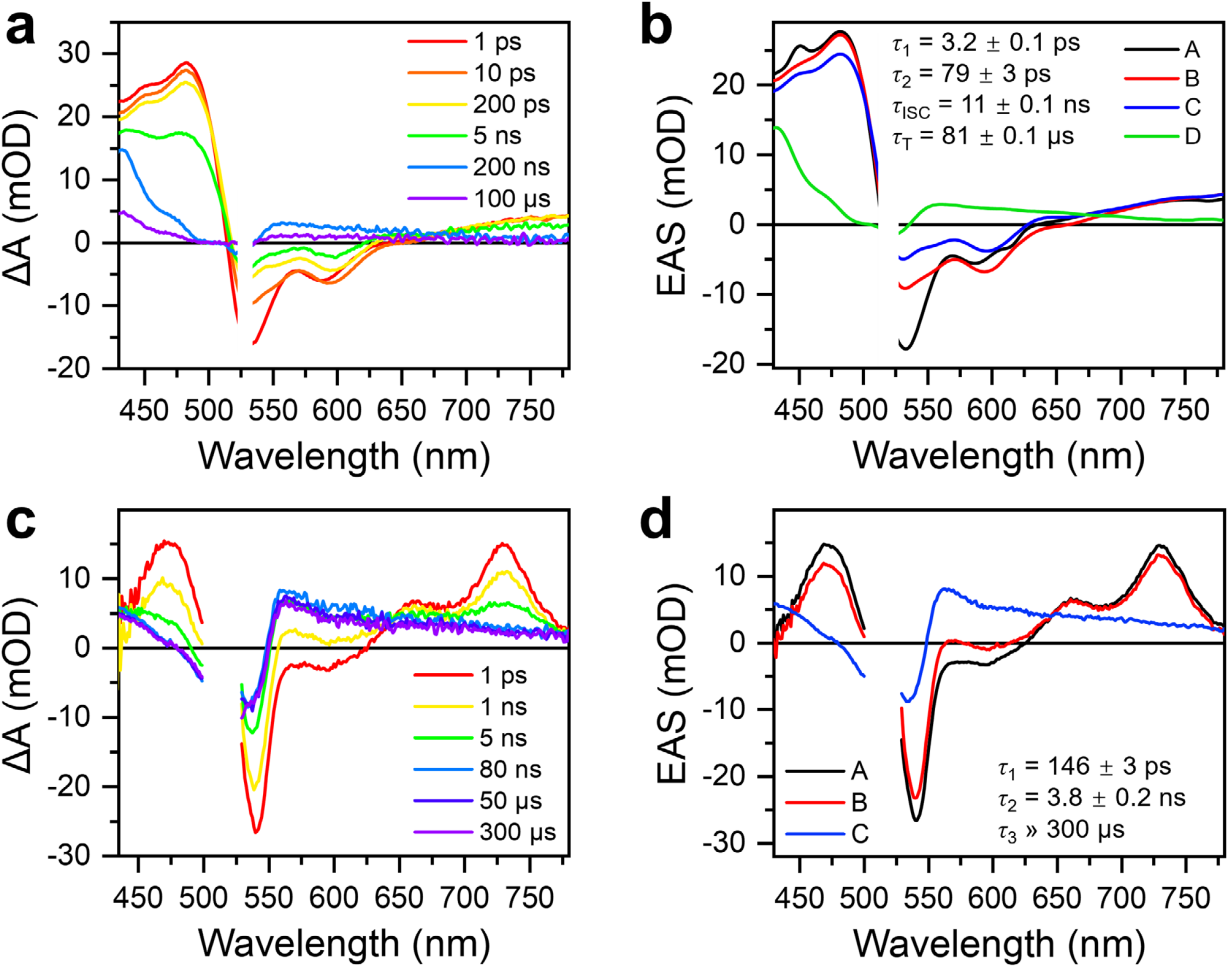
(a), (b) Transient absorption spectra at selected pump–probe delay times and the corresponding evolution‐associated spectra (EAS) of Am‐NDI in air‐free toluene at room temperature (ex: 520 nm, 2 mJ/cm^2^). (c), (d) Transient absorption spectra and EAS of Am‐NDI‐doped NDI crystal at 85 K (ex: 520 nm, 3 mJ/cm^2^).

Transient absorption (TA) measurements were conducted on Am‐NDI in degassed toluene to probe the excited‐state dynamics (Figure 3a). At room temperature, excitation at 520 nm results in a broad negative signal between 510 nm and 650 nm, attributed to overlapping ground‐state bleach (GSB) and stimulated emission (SE). Concurrently, excited‐state absorption (ESA) bands are observed at 420 and 750 nm. The observed temporal evolution of the TA signals suggests the presence of multiple excited‐state species. To resolve their individual spectral signatures, global analysis was performed using a sequential A → B an→ C → D → ground state kinetic model, yielding the evolution‐associated spectra (EAS) shown in Figure 3b. The EAS exhibit nearly identical spectral shapes at early times, indicating that relaxation processes occur within the same electronic state. Therefore, the first relaxation (A → B) with a time constant of ∼3.2 ps is attributed to solvent relaxation within the excited singlet state (S_1_), which induces a slight red shift (∼7 nm) of the SE feature. The subsequent relaxation process (B → C) proceeds more slowly (∼79 ps) and is assigned to a small structural relaxation. The S_1_ state undergoes ISC to the triplet state (T_1_) with a time constant of 11 ns. The T_1_ state is characterized by a strong absorption band around 430 nm [41] and a broad band spanning 530–780 nm and subsequently decays to the ground state (*S*_0_) with a lifetime of ∼81 µs.

The inert electronic environment of the host lattice suggests that Am‐NDI undergoes triplet formation in the solid state, analogous to its behavior in solution. To test this, TA spectra were collected on Am‐NDI‐doped single crystals at 85 K using 520 nm excitation, which selectively excites the ICT band of Am‐NDI (Figures 3 and S6). The pump and probe beams were linearly polarized, parallel to each other, and aligned with the crystallographic *a‐*axis. Global analysis of the spectral evolution was performed using an A → B → C → ground state kinetic model (Figure 3d). Following photoexcitation, the initially formed singlet excited state (species A) exhibits a GSB at 542 nm and SE at 600 nm, along with ESA bands spanning 425–510 nm and 630–780 nm. In the crystal, the relative intensity of the ESA band in the 630–780 nm region compared to that in the 425–510 nm region of the S_1_ spectrum is higher than in solution, as also observed for the T_1_ state (species C). This difference may arise from the fixed molecular orientation in the crystal, which leads to anisotropic ESA, and from differences in the local environment of AmNDI in solution and the single crystal. Species A relaxes to species B in *τ*_A→B_ = 146  ±  3 ps, a structural relaxation that proceeds more slowly than in solution owing to the rigid crystal lattice and lower temperature. Intersystem crossing from species B to the triplet state (species C, ^3^Am‐NDI) occurs in 3.8  ±  0.2 ns, as also confirmed by time‐resolved fluorescence measurements, which reveal a single emissive species (Figure S7). This process produces ^3^Am‐NDI with 52% yield, estimated from the ratio of the initial GSB to that of the triplet exciton at long delay times (Figure S8) [42]. The ^3^Am‐NDI state subsequently decays to *S*_0_ with a lifetime much longer than 300 µs, confirming the formation of a long‐lived, localized triplet state in the doped crystal.

### 2.3 Single‐Crystal Time‐Resolved EPR Spectroscopy

**FIGURE 4:**
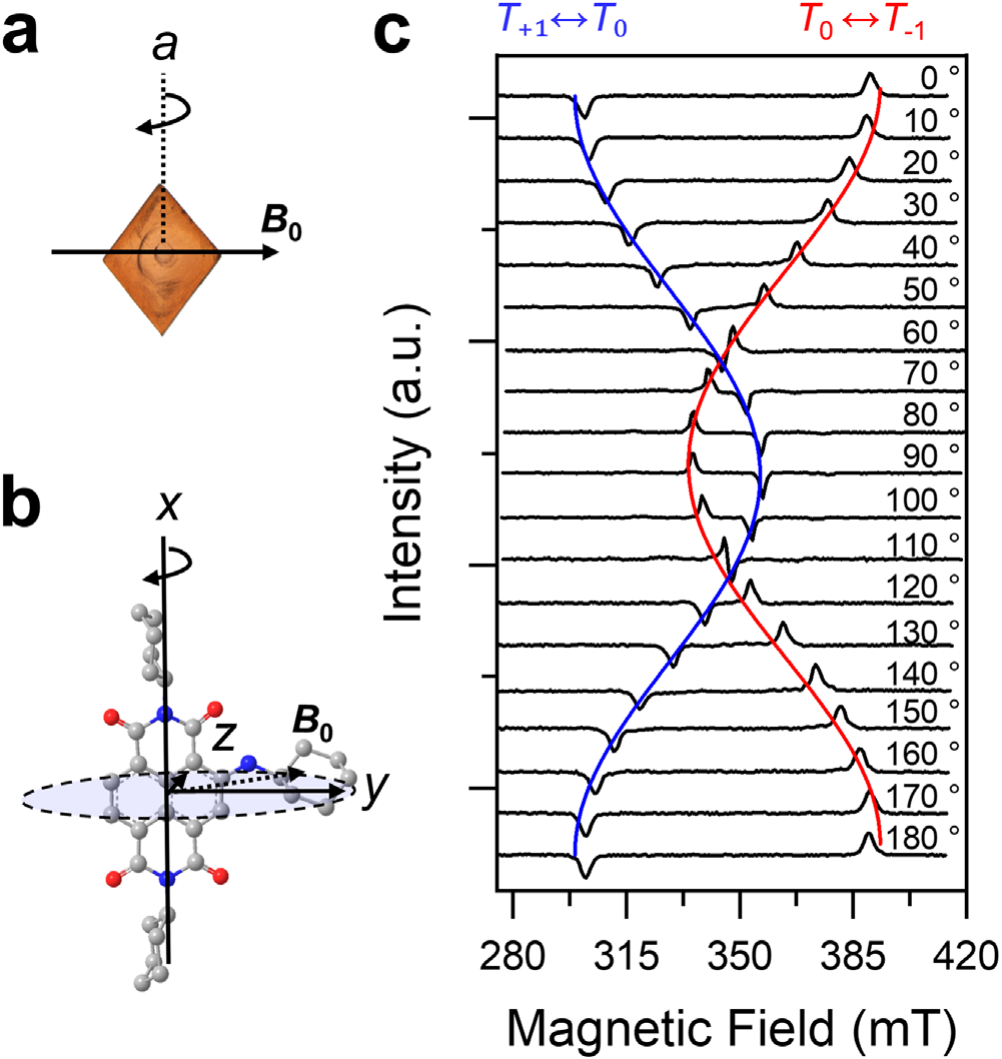
(a) A representative image showing the alignment of a doped single crystal within the laboratory frame, with the directions of the magnetic field and rotation axis indicated relative to the crystal axes. (b) Illustration of the orientation of the applied magnetic field relative to the molecular coordinate axes of Am‐NDI. (c) Orientation‐dependent TREPR spectra of the Am‐NDI doped NDI single crystal at 85 K and 150 ns after 520 nm excitation, with the crystal rotated from *θ* = 0°–180° in ∼10° steps, where *θ* is defined as the angle between the external magnetic field and the molecular *z*‐axis within the *yz* plane (*θ* = 0° corresponds to the field parallel to the molecular *z*‐axis). Simulated *T*_+__1_ ↔ *T*_0_   and *T*_0_ ↔ *T*_−__1_ transitions are shown in blue and red, respectively.

As previously discussed, triplet excitons are generated via the ISC mechanism in the doped single crystals at 85 K upon 520 nm photoexcitation. This process typically produces a non‐Boltzmann population of the triplet sublevels, which was characterized by TREPR spectroscopy (Figure 4). Moreover, unlike in glassy solids or polycrystalline powders, where molecules are randomly oriented, those in single crystals adopt a well‐defined orientation due to molecular packing. This leads to anisotropic alignment of triplet excitons, with their zero‐field splitting (ZFS) tensors fixed relative to the crystallographic axes. As a result, rotation of the crystal with respect to the external magnetic field produces narrow EPR transitions at distinct resonance fields.

As shown in Figures 4a and b, the doped single crystal was mounted in the EPR spectrometer and rotated around its crystal *a*‐axis, which is parallel to the molecular *x*‐axis of Am‐NDI, as determined by x‐ray face indexing (Figure S4). This configuration corresponds approximately to a rotation of the applied magnetic field within the *yz* plane of the ZFS tensor (Figure S10). As shown in Figure 4c, orientation‐dependent TREPR spectra were collected in 10° increments. At each rotation angle, only two peaks—either (*e*,*a*) or (*a*,*e*)—were observed from low to high magnetic field, where “*a*” denotes enhanced absorption and “*e*” denotes emission. These peaks arise from the *T*_+1_ ↔ *T*_0_ (emission, blue) and *T*_−1_ ↔ *T*_0_ (absorption, red) transitions, with their angular dependence captured in the fitted trace. Assuming the ZFS parameter *D* > 0, the unchanged signal polarity across all angles suggests spin‐selective population of the *T*_x_ and *T*_z_ sublevels. This spin polarization is consistent with the (*eea*, *eaa*) TREPR pattern observed in doped crystal powders (Figure S9), where the relative populations of the triplet sublevels are *P*_x_ = 0.9, *P*_y_ = 0, and *P*_z_ = 0.1 (*T*_x_, *T*_y_, and *T*_z_, respectively). The pattern can be fitted using the same *D* and *E* parameters as those obtained in toluene glass at 85 K (*D* = 1320 ± 20 MHz, *E* = ‐416 ± 6 MHz), confirming that the triplet excitons are localized on the Am‐NDI. These *D* and *E* parameters also reproduce the orientation‐dependent TREPR spectra of the doped single crystal, with the simulated curves matching the experimental data. Full simulations of the angle‐dependent TREPR spectra are shown in Figure S10. (see the fitting details in Supporting Information). This agreement further indicates that Am‐NDI replaces NDI molecules while preserving the same molecular alignment within the doped crystal lattice.

### 2.4 Temperature‐Dependent Spin Decoherence

**FIGURE 5:**
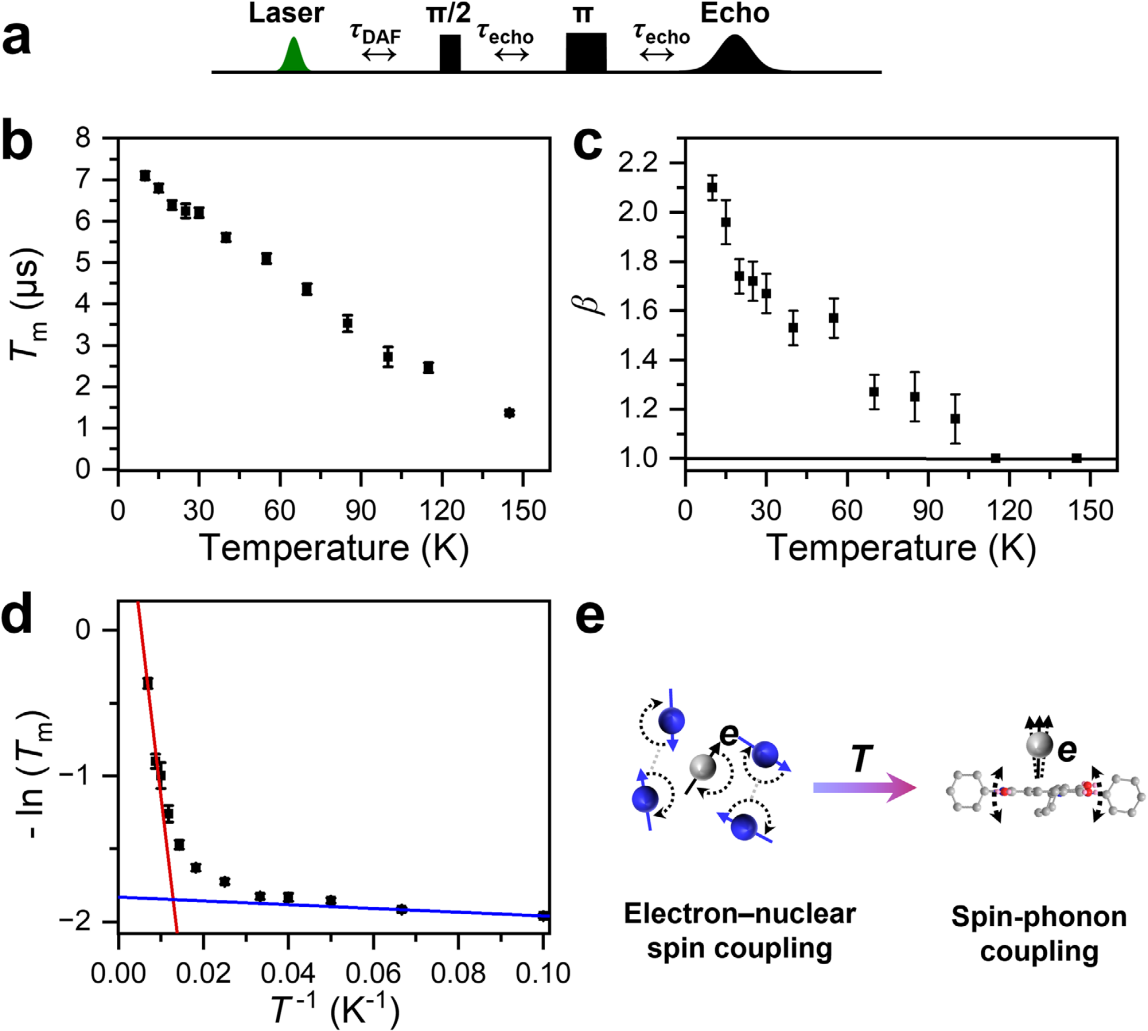
(a) Hahn echo sequence used to measure coherence times following photoexcitation, with the inter‐pulse delay *τ*_echo_ incrementally varied. (b) Spin coherence times of triplets at *θ* = 0° in Am‐NDI doped NDI single crystals measured at various temperatures (*τ*_DAF_ = 1.4 µs). (c) Temperature dependence of the stretching exponent *β* obtained from fitting the coherence decay traces using a stretched exponential function. (d) Arrhenius plot of −ln(*T*_m_) versus *T* ^−1^, showing two distinct regimes. The high‐temperature region (red line) follows thermally activated behavior with an activation energy of 170 ± 17 cm^−1^, while the low‐temperature region (blue line) shows weak temperature dependence. (e) Schematic illustration of two key decoherence mechanisms in Am‐NDI doped NDI crystals. Left: at low temperatures, spin dephasing dominated by electron–nuclear spin coupling. Right: at elevated temperatures, spin dephasing dominated by spin–phonon coupling, where phonons modulate spin Hamiltonian parameters such as the hyperfine‐, *g*‐, or *D*‐tensors.

As previously discussed, doped single crystals provide intrinsic advantages for preserving spin coherence. These advantages arise from suppression of exciton hopping and from reduction of electron dipolar coupling, the latter achieved through using low doping concentrations that minimize spin density. To experimentally validate these anticipated advantages and further investigate spin coherence in this system, we characterized the spin coherence time by variable‐temperature pulse‐EPR spectroscopy using a two‐pulse Hahn echo sequence (Figure 5a), in which a π/2 pulse is followed by a π pulse that refocuses dephasing spins. The resulting spin echo decay as a function of inter‐pulse delay provides a direct measure of spin coherence lifetime. Temperature‐dependent measurements were performed on Am‐NDI‐doped NDI single crystals at *θ* = 0°, using a fixed delay after photoexcitation (*τ*_DAF_ = 1.4 µs). The echo intensity as a function of 2*τ* at various temperatures is shown in Figure S11. Oscillations were observed in the echo decay dynamics, arising from electron–nuclear hyperfine coupling with nitrogen‐14 (^14^N) and hydrogen (^1^H) nuclei, as confirmed by Fourier transform analysis at both X‐band and Q‐band (Figure S12). The decays were fitted using either a stretched or mono‐exponential function, and the extracted *T*_m_ values are summarized in Figure 5b.

As shown in Figure 5b, *T*_m_ reaches 7.1 µs at 10 K, comparable to values reported for triplet excitons in donor‐acceptor cocrystals [16, 17, 19]. Notably, it remains 3.5 µs at 85 K and 1.4 µs at 145 K which exceed those cocrystals within the liquid‐nitrogen temperature regime [16, 17, 19]. Detailed Arrhenius analysis of the decoherence rate, plotted as 1/*T*_m_ versus 1/*T* (K^1^) in Figure 5c, resolves two linear regimes. The low‐temperature region exhibits only a very weak temperature dependence with a negligible activation energy, whereas the higher‐temperature regime shows a strong dependence with an activation energy (170 ± 17 cm^−1^). This behavior indicates a crossover in the dominant decoherence mechanism between the low‐ and high‐temperature ranges. At low temperatures, where most of motional degrees of freedom are frozen, decoherence is usually governed by electron–electron dipolar coupling [17, 19, 26] and electron‐nuclear hyperfine interactions [16, 43, 44, 45]. Since dipolar interactions scale with the density of spins in the crystal, we examined the excitation‐fluence dependence of *T*_m_ at 20 K (Figure S13). The absence of any variation in *T*_m_ over the tested fluence range indicates that electron–electron dipolar coupling does not dominate decoherence in this regime. This takes advantage of the dilute doping level (0.1%) in our single crystal, where the estimated average distance between dopant molecules (∼8 nm) minimizes electron–electron dipolar coupling and thus suppresses decoherence.

Instead, as shown in Figure 5c, when *T* ≤ 20 K, the echo decay is best described by a stretched exponential function with *β* ≈ 2: $I=I_{0}+Ae^{{\left(\frac{-2\tau }{T_{2}}\right)}^{\beta }}$, where *I* is the echo intensity as a function of the interpulse delay *τ*, *I*_0_ is the extrapolated echo intensity at zero delay, and *β* is the stretch factor, which depends on the underlying dephasing mechanism [46]. This is a hallmark of spectral diffusion driven by nuclear spin flip–flop processes which arise from dipolar interactions within the nuclear spin bath. When nuclei exchange their spin states through a flip‐flop process, the spatial distribution of nuclear polarization changes, leading to fluctuations in the effective hyperfine field experienced by the electron spins and ultimately leading to spin decoherence (Figure 5e, left)[46, 47, 48]. Nuclear spin flip–flops are energy‐conserving mutual spin exchange process, and are therefore not thermally activated, as evidenced by the nearly temperature‐independent trend represented by the blue fit in Figure 5d. This nuclear spin–driven decoherence is particularly pronounced owing to the high nuclear spin density in the crystal lattice. It has been shown that reducing the concentration of nuclear spins by deuteration can significantly reduce this decoherence and thus markedly prolong electron spin coherence times [16, 26, 44, 49].

As the temperature increases, however, *T*_m_ gradually decreases, accompanied by a decreasing *β*. This trend suggests the emergence of an additional decoherence pathway. Above 115 K, the decay curves can be adequately fitted with a monoexponential function, indicating that a different decoherence mechanism dominates. Previous reports have shown that, at elevated temperatures, thermally activated processes such as exciton hopping and spin‐phonon coupling become the primary sources of decoherence [16, 17, 18, 19]. Given that the triplet exciton is localized on the dopant, thermally activated dopant‐to‐host triplet transfer could deplete the triplet population and thereby limit *T*_m_. To examine this possibility, the triplet population lifetime (*T*_pop_) was determined by nanosecond transient absorption (nsTA) spectroscopy over 85–145 K (Figure S14). A slight decrease in *T*_pop_ with temperature was observed up to ∼120 K, while pronounced thermal activation occurred at higher temperatures. Arrhenius analysis yields an activation energy *E_a_* of 198 ± 2 meV, matching the triplet‐energy offset between the dopant and host (Figure S2) and consistent with dopant‐to‐host triplet transfer. Despite this decrease, *T*_pop_ remains more than two orders of magnitude longer than *T*_m_ across the entire range, indicating that population decay is not the limiting factor for spin coherence (Figure S16).

Spin‐phonon coupling contributes to decoherence through two distinct pathways: energy relaxation (*T*_1_) via phonon‐assisted spin flips, which consequently limits *T*_m_, and pure dephasing, which directly shortens *T*_m_ by modulating spin Hamiltonian parameters and generating local magnetic field fluctuations (Figure 5e, right) [50]. The influence of spin‐phonon coupling becomes stronger with increasing temperature, as the phonon population rises steeply following the Bose–Einstein distribution. First, we measured the spin‐polarization lifetime (*T*_pol_) obtained from echo‐detected intensities as a function of *τ*_DAF_ (Figures S15). Because the population lifetime *T*_pop_ » *T*_pol_, the *T*_pol_ predominantly reflects the spin‐lattice relaxation and can therefore be approximated as *T*_1_. *T*_pol_ times are approximately an order of magnitude longer than *T*_m_, further precluding a *T*_1_‐limited *T*_m_ regime. Hence, we attribute the spin decoherence observed at elevated temperatures to the phonon‐assisted modulation of anisotropic spin tensors, such as the hyperfine‐, *g*‐ or *D*‐tensors for the triplet excitons. In organic materials, the anisotropic spin tensors are particularly susceptible to perturbations from molecular vibrations [50, 51, 52] and rotations [53], which are strongly promoted by the intrinsic flexibility of the molecular skeleton and the relatively soft lattice framework. Moreover, a phonon mode at ∼148 cm^1^ was recently observed in perylenediimide–based donor–acceptor cocrystals and assigned to in‐plane rotational motions that perturb ZFS tensors thus mediate decoherence [16]. In this work, the activation energy of ∼170 cm^1^ extracted from the temperature dependence of *T*_m_ (at *Z* transition), falls within the range of low‐frequency vibrational and rotational modes that may involve out‐of‐plane molecular motions contributing to decoherence.

## 3 Conclusions

In summary, we engineered a new doped single crystal using a host‐derived molecule as the dopant, which sustains long spin coherence times at elevated temperatures. Polarization‐dependent absorption together with single‐crystal TREPR measurements confirm the effectiveness of this dopant strategy, showing that the dopant molecules are incorporated into the host lattice and adopt the same orientation as the host molecules. The ICT character of the dopant not only allows for its selective excitation but also facilitates efficient triplet localization at the dopant sites. Transient absorption measurements unambiguously reveal the formation of triplet excitons via ISC with a 52% quantum yield. Moreover, pulse‐EPR measurements show that the spin coherence of triplets reaches 7.1 µs at 10 K and 3.5 µs at 85 K and remains as long as 1.4 µs at 145 K, demonstrating that qutrits photogenerated in organic crystal systems can operate in the liquid‐nitrogen temperature regime. Various temperature‐dependent measurements were performed to investigate the decoherence mechanisms. We found that, although electron–electron dipolar coupling and exciton hopping can be suppressed, nuclear spin flip–flops and spin‐phonon coupling impose fundamental limits on *T*_m_. Spin coherence is strongly limited by nuclear spins owing to the dense packing and high proton density in organic crystals. This limitation could be substantially mitigated by deuteration. At elevated temperatures, the spin–phonon effect becomes inevitably pronounced, owing to perturbations of the anisotropic spin tensors by phonons arising from molecular motions. Based on these results and comparisons with one of the most mature doped crystal systems, pentacene‐doped terphenyl crystals, where the greater molecular rigidity of pentacene may contribute to its longer coherence lifetime, future efforts will focus on designing more rigid ICT‐based dopants to further suppress these decoherence channels. Overall, this study provides valuable guidance for the rational design of doped organic crystal systems aimed at achieving prolonged triplet exciton coherence lifetimes to serve as qutrits for quantum technologies.

## Conflicts of Interest

The authors declare no conflicts of interest.

## Supporting information




**Supporting File 1**: anie71793‐sup‐0001‐SuppMat.pdf.

**Supporting File 2**: anie71793‐sup‐0002‐Data.zip.

## Data Availability

The data that support the findings of this study are available in the Supporting Information of this article.
