# Supplementary material for: Intramolecular Charge‐Transfer Dopants Enable Isolated Triplet Excitons as Spin Qutrits in a Single Crystal
Source: Angew Chem Int Ed Engl. 2026 Mar 9;65(17):e24670. doi: 10.1002/anie.202524670 (PMC13098311; doi:10.1002/anie.202524670)
Supplement: Supplementary file 1 — Supporting File 1: anie71793‐sup‐0001‐SuppMat.pdf. [file ANIE-65-e24670-s002.pdf]

Supporting Information for

Intramolecular Charge-Transfer Dopants Enable Isolated Triplet Excitons as Spin Qutrits in a Single Crystal

Yaoyao Han, Samuel B. Tyndall, Kathryn R. Peinkofer, Yuheng Huang,

Ryan M. Young*, Matthew D. Krzyaniak*, and Michael R. Wasielewski*

Department of Chemistry, Institute for Quantum Information Research and Engineering, and

Center for Molecular Quantum Transduction, Northwestern University,

Evanston, IL 60208-3113, United States

*Correspondence to:m-wasielewski@northwestern.edu,

mdkrzyaniak@northwestern.edu, ryan.young@northwestern.edu

Table of Contents

1. Synthesis 2

2. Single-Crystal Growth and Structure Determination. 3

3. Steady-State Optical Characterization. 7

Steady-State Absorption Spectroscopy 7

Steady-State Phosphorescence Spectroscopy 7

4. Time-resolved spectroscopy. 8

Transient Absorption Spectroscopy. 8

Time-Resolved Fluorescence Spectroscopy. 8

5. Electron Paramagnetic Resonance (EPR) Spectroscopy. 9

Sample preparation 9

Time-Resolved EPR Spectroscopy. 9

Pulse-EPR Spectroscopy. 10

6. Supporting Figures 11

7. References 17

1. Synthesis

All chemicals were purchased from the chemical suppliers and used without further purification. Naphthalene-1,4:5,8-tertacarboxy dianhydride was purchased from TCI. Cyclohexanamine was purchased from Thermo Fisher Scientific. Zinc acetate was purchased from Sigma-Aldrich. Imidazole was purchased from Alfa Aesar.

The ^{1}H-NMR spectra were collected on a Bruker Avance III 500 MHz system w/ DCH cryoprobe (ag500) or w/ Prodigy probe (x500) at the Integrated Molecular Structure Education and Research Center (IMSERC) at Northwestern University. The mass spectrometry was performed on a Bruker AutoFlex III Smartbeam or Bruker RapiFlex Tissue Typer matrix assisted laser desorption ionization–time of flight (MALDI-TOF) system at IMSERC at Northwestern University.

NDI was synthesized using previously published procedures.[1] ^{1}H-NMR (500 MHz, $CDCl_3$): δ= 8.63 (s, 4H), 4.90-4.98 (m, 2H), 2.40-2.5 (m, 4H), 1.85 (d, 4H), 1.68 (m, 6H), 1.33-1.44 (m, 4H)1.20-1.31 (m, 2H). MS (MALDI-TOF) m/z: calculated for $C_{26}H_{26}N_2O_4$, $[M]^+$ 431.19; found 431.57.

Am-NDI was synthesized following the method outlined in Figure S1.

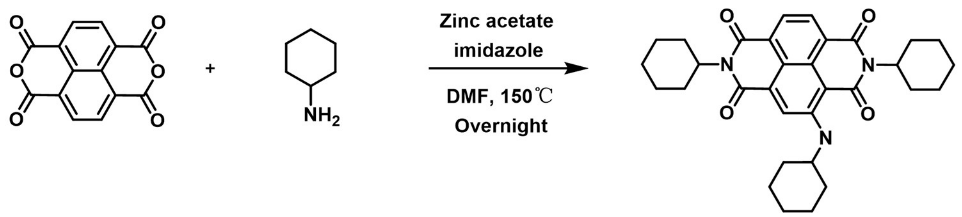

Figure S1. Synthetic procedure of **Am-NDI**.

A mixture of 1,4,5,8-naphthalenetetracarboxylic dianhydride (NDA, 250 mg, 0.93 mmol), cyclohexylamine (10 mL, 86 mmol), imidazole (17 mg, 0.25 mmol), and zinc acetate (5 mg, 0.027 mmol) in 30 mL of DMF was heated at 150 °C overnight. The crude product was purified by column chromatography using hexanes/ethyl acetate (10:1) as the eluent, affording Am-NDI as a red solid (10 mg, 2%).^{1}H-NMR (500 MHz, $CDCl_3$): δ= 10.14 (d,1H), 8.53 (d, 1H), 8.21 (d,1H), 8.11 (s, 1H), 4.86-5.03 (m, 2H), 3.76 (s, 1H), 2.38-2.57 (m, 4H), 2.13 (m, 2H), 1.76-1.88 (m, 6H), 1.60-1.71 (m, 7H), 1.32-1.55 (m, 11H). MS (MALDI-TOF) m/z: calculated for $C_{32}H_{37}N_3O_4$, $[M]^+$ 528.28; found 528.63.

2. Single-Crystal Growth and Structure Determination.

NDI single crystals were grown by slow vapor diffusion at room temperature. In a typical procedure, NDI was dissolved in dichloromethane (DCM) to give a ~3.7 mM solution, which was filtered through a 0.2 μm PTFE filter. Methanol vapor was allowed to diffuse into this solution over 4–6 days, yielding pale yellow NDI crystals. The same procedure was followed for preparing Am-NDI-doped NDI crystals, except that Am-NDI was added to the NDI solution at a concentration of ~3.6 μM. Slow vapor diffusion of methanol into the Am-NDI/NDI solution over 4–6 days produced orange doped crystals**.** The final doping concentration (~0.1 %) was determined by dissolving the doped crystals, measuring the absorbance of the resulting solution using UV–vis spectroscopy, and calculating the concentration based on the molar extinction coefficients of NDI and Am-NDI.

A single crystal was mounted on a MiTeGen loop using paratone oil and placed on an XtaLAB Synergy diffractometer equipped with micro-focus rotating-anode X-ray tube Rigaku (Cu) X-ray

source and a Hybrid Pixel Array Detector (HyPix) detector. The crystal was cooled to ~100 K with an Oxford cryostat for data collection. CrysAlisPro was used for face indexing, data integration, and empirical absorption correction. The structure was solved by intrinsic phasing with ShelXT in Olex2. Refinement was carried out using ShelXL and least-squares minimization. Final CIFs have been deposited with the CCDC under numbers 2488462 (undoped NDI single crystal) and 2487488 (doped single crystal). Complete crystallographic details are provided in Tables S1–S2.

Table S1. Single crystal data and structure refinement for NDI.

Empirical formula	$C_{26}H_{26}N_2O_4$
Formula weight	430.49
Temperature/K	100.00(10)
Crystal system	monoclinic
Space group	$P2_1/c$
a/Å	18.2950(4)
b/Å	6.6293(2)
c/Å	8.4124(2)
α/°	90
β/°	101.719(2)
γ/°	90
Volume/Å^3	999.01(4)
Z	2
ρcalcg/cm^3	1.431
μ/mm-1	0.784
F(000)	456.0
Crystal size/mm^3	1.43 × 1.12 × 0.44
Radiation	Cu Kα (λ = 1.54184)
2Θ range for data collection/°	14.246 to 150.132
Index ranges	-22 ≤ h ≤ 22, -8 ≤ k ≤ 8, -7 ≤ l ≤ 10
Reflections collected	6037
Independent reflections	1997 [Rint = 0.0151, Rsigma = 0.0116]
Data/restraints/parameters	1997/0/145
Goodness-of-fit on F2	1.064
Final R indexes [I>=2σ (I)]	R1 = 0.0418, wR2 = 0.1320
Final R indexes [all data]	R1 = 0.0430, wR2 = 0.1333

Table S2. Single crystal data and structure refinement for 0.1% Am-NDI doped into NDI.

Empirical formula	$C_{26}H_{26}N_2O_4$
Formula weight	430.49
Temperature/K	100.00(10)
Crystal system	monoclinic
Space group	$P2_1/c$
a/Å	18.2931(8)
b/Å	6.6313(3)
c/Å	8.4082(3)
α/°	90
β/°	101.696(4)
γ/°	90
Volume/Å^3	998.80(7)
Z	2
ρ_{calc}g/cm^3	1.431
μ/mm^{-1}	0.784
F(000)	456.0
Crystal size/mm^3	1.61 × 1.23 × 0.26
Radiation	Cu Kα (λ = 1.54184)
2Θ range for data collection/°	9.876 to 157.868
Index ranges	-23 ≤ h ≤ 23, -8 ≤ k ≤ 8, -10 ≤ l ≤ 4
Reflections collected	6384
Independent reflections	2085 [R_{int} = 0.0286, R_{sigma} = 0.0238]
Data/restraints/parameters	2085/0/146
Goodness-of-fit on F^2	1.072
Final R indexes [I>=2σ (I)]	R_1 = 0.0462, wR_2 = 0.1459
Final R indexes [all data]	R_1 = 0.0489, wR_2 = 0.1484

3. Steady-State Optical Characterization.

Steady-State Absorption Spectroscopy.

Solution samples: samples were dissolved in DCM and transferred to 2 mm quartz cuvettes. UV−vis absorption spectra of solution samples were obtained using a Shimadzu UV-1800 spectrometer.

Crystal samples: crystal samples were mounted on glass coverslips using a UV-curable epoxy (IllumaBond 60−7180RCL16, Epoxies, Etc.) for measurements. Polarization-resolved steady-state absorption spectra were collected on single crystals as a function of linear polarization of the incident light (0−90° in 10° steps) using an optical microscope described previously.[2]

Steady-State Phosphorescence Spectroscopy.

Solution samples were prepared in 2-methyltetrahydrofuran (mTHF) under a N_2 atmosphere in a glovebox, loaded into quartz tubes, and sealed with Parafilm. The tubes were rapidly transferred to a glass Dewar flask and cooled to 77 K prior to measurement. Phosphorescence spectra were recorded on a Horiba Nanolog spectrofluorimeter (FL3-2iHR/iHR) using flash lamp excitation with time-gated detection (gate delay = 2 ms), which enables temporal separation of prompt fluorescence from phosphorescence.

4. Time-resolved spectroscopy.

Transient Absorption Spectroscopy.

The femtosecond (fsTA) and nanosecond (nsTA) transient absorption spectroscopy setups and data acquisition methods have been reported previously.[3] Solution samples for TA experiments were prepared in toluene, loaded into 2 mm quartz cuvettes, and degassed using a freeze–pump–thaw method. Doped crystal samples were mounted on glass coverslips using a UV-curable epoxy (IllumaBond 60−7180RCL16, Epoxies, Etc.). For TA experiments at low temperatures, the mounted crystal was placed inside a Janis VNF-100 cryostat (Janis Research Co. LLC) coupled to a Cryo-Con 32B (Cryogenics Control Systems, Inc.) temperature controller. The sample was then cooled to desired temperatures to measure the TA spectra. The linear polarizations of the pump and probe beams were controlled using achromatic half-wave plates and aligned along the long axis of the crystal. Excitation was performed at 520 nm with pulse energies of 0.5–1 μJ, focused to a spot size of ~0.2 mm in diameter, resulting in a pump fluence of 1.6–3 mJ/cm². Kinetic analysis was performed using lab-written programs in MATLAB.

Time-Resolved Fluorescence Spectroscopy.

Time-resolved fluorescence (TRF) measurements were performed using a commercial diode-pumped 100 kHz laser (Spirit 1040-HE, Spectra-Physics), producing a 1040 nm fundamental beam (~350 fs, 12 W), which was used to pump a noncollinear optical parametric amplifier (Spirit NOPA, Spectra-Physics) to generate 500 nm excitation pulses that were used to

excite the doped crystals. Fluorescence signals were collected with a Hamamatsu C4780 Streakscope, as reported previously.[4] Data acquisition was conducted in single-photon-counting mode using the Hamamatsu HPD-TA software. The temporal resolution corresponded to approximately 2% of the sweep window. Samples were mounted on glass coverslips using a UV-curable epoxy.

5. Electron Paramagnetic Resonance (EPR) Spectroscopy.

Sample preparation.

An Am-NDI–doped NDI single crystal was mounted on a cut glass coverslip in the desired orientation and fixed with ultraviolet-curable epoxy. The coverslip was then transferred into a quartz EPR tube (2.4 mm o.d., 2.0 mm i.d.) and further secured in place with ultraviolet-curable epoxy.

Time-Resolved EPR Spectroscopy.

Time-Resolved EPR measurements were performed at X-band (~9.5 GHz) using a Bruker Elexsys E680 X/W EPR spectrometer and a split-ring resonator (ER4118X-MS3). The temperature was maintained at the desired setpoints using an Oxford Instruments CF935 continuous-flow cryostat, cooled with either liquid nitrogen or helium. Samples were photoexcited using 7 ns, 520 nm laser pulses generated by an optical parametric oscillator (OPO, Spectra-Physics BasiScan), pumped by the 355 nm output of a 10 Hz Nd:YAG laser (Spectra-Physics

Quanta-Ray Lab-170-10H). The laser light was coupled into the resonator via a fiber optic and collimator positioned outside the cryostat. Laser power was measured directly at the fiber-optic output.

Data processing and spectral fitting were performed in MATLAB with custom scripts and EasySpin v6.0.6. Triplet TREPR spectra were simulated and fit using the *pepper* function in EasySpin according to the spin Hamiltonian

$$\hat{\mathcal{H}} = \mu_B g \boldsymbol{B} \cdot \boldsymbol{S} + D\left(\hat{S}_z{}^2 - \frac{S(S+1)}{3}\right) + E\left(\hat{S}_x{}^2 - \hat{S}_y{}^2\right) \qquad (1)$$

where μ_B is the Bohr magneton, g is the isotropic g-factor, $\boldsymbol{B}$ is the magnetic field vector, $\boldsymbol{S}$ is the vector of triplet spin operators ($\hat{S}_x,\ \hat{S}_y,\ \hat{S}_z$) is the vector containing the triplet spin operators, S is the total spin (S = 1), and D and E are the axial and rhombic zero field splitting parameters, respectively.

Pulse-EPR Spectroscopy.

All pulse-EPR data were acquired in quadrature using an overcoupled resonator (Q < 200). Coherence times (T_m) were determined using a two-pulse Hahn-echo sequence, where the delay time between the π/2 (16 ns)and π (32 ns)microwave pulses, τ_{echo}, was varied and the delay time between photoexcitation and the π/2 microwave pulse, τ_{DAF}, was fixed at 1.4 μs. Echo signals were integrated over their full width at half-maximum (FWHM) and plotted versus 2τ_{echo}. The population lifetime was determined using same sequence, with τ_{echo} = 200 ns and τ_{DAF} was varied.

6. Supporting Figures

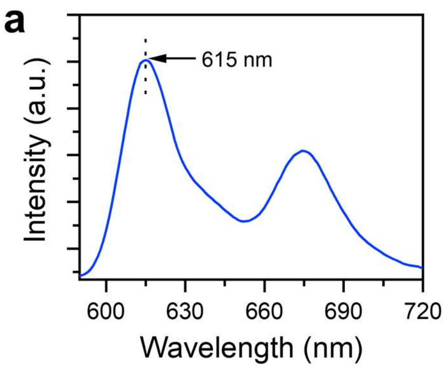

Figure S2. Phosphorescence spectra of (a) NDI excited at 380 nm and (b) Am-NDI excited at 510 nm in *m*-THF at 77 K. Triplet energies determined from the first vibronic peak are 2.02 eV (NDI) and 1.83 eV (Am-NDI), differing by 190 meV.

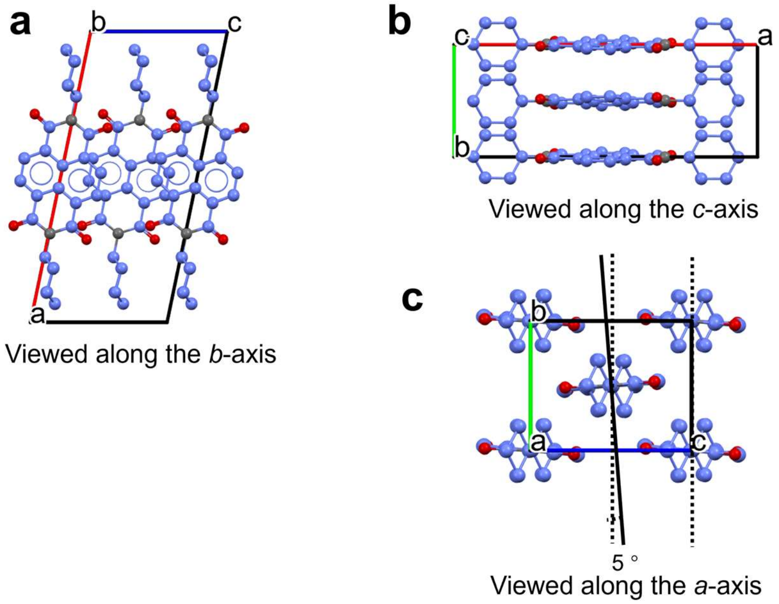

Figure S3. Crystal structure of NDI crystals viewed along the (a) *b*-, (b) *c*-, and (c) *a*- axes. Carbon, nitrogen, and oxygen atoms are shown in blue, dark gray, and red, respectively. Hydrogen atoms are omitted for clarity. Misalignment angles between the molecular axes of non-translationally equivalent NDI molecules within the unit cell are illustrated in (c).

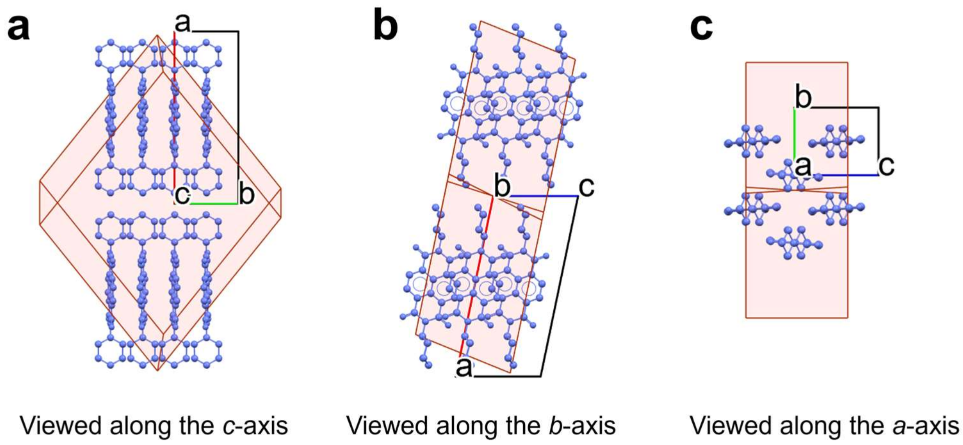

Figure S4. Face-indexed crystal overlaid with the unit cell and viewed along (a) the *c*-axis; (b) the *b*-axis (c) the *a*-axis

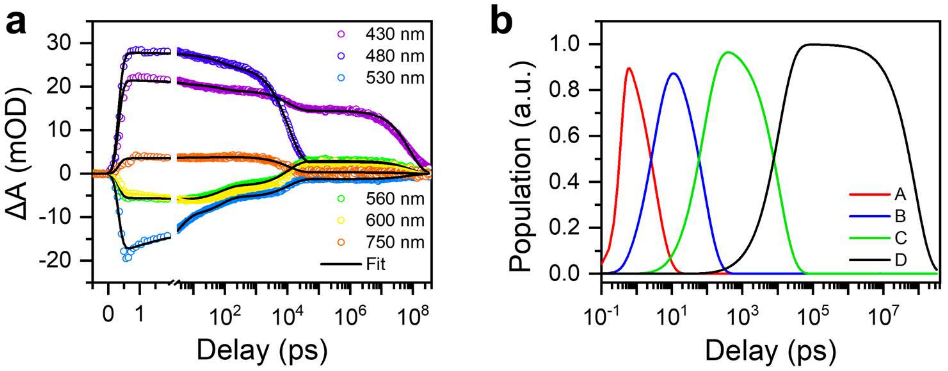

Figure S5. (a) Representative kinetic traces of Am-NDI in air-free toluene at selected wavelengths at room temperature (ex: 520 nm, 2 mJ/cm^2). (b) Time-dependent population dynamics obtained from kinetic modeling.

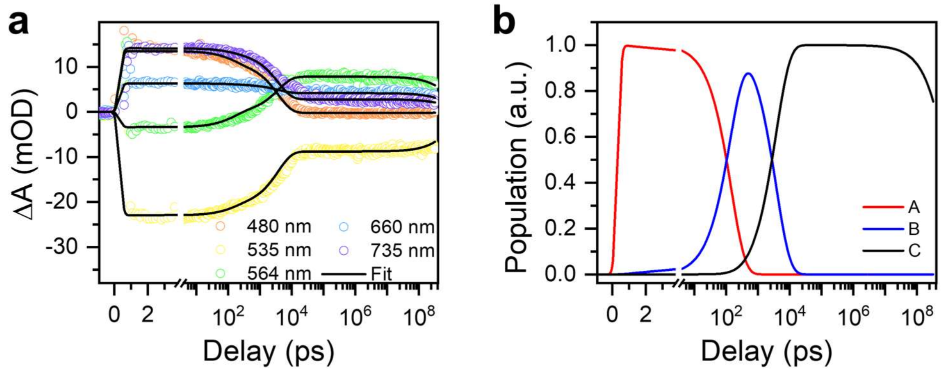

Figure S6. (a) Representative kinetic traces of an 0.1% Am-NDI-doped NDI single crystal at selected wavelengths at 85 K (ex: 520 nm, 3 mJ/cm^2). (b) Time-dependent population dynamics obtained from kinetic modeling.

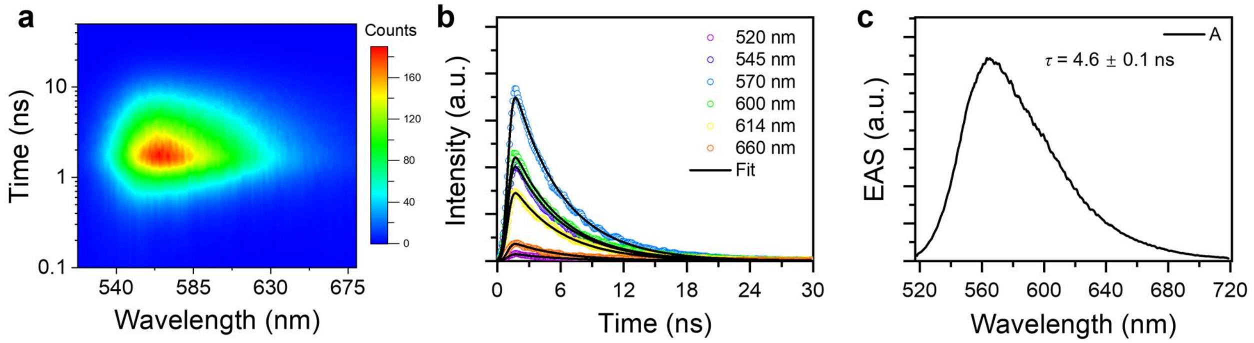

Figure S7. Time-resolved fluorescence of 0.1% Am-NDI-doped NDI crystals recorded under 500 nm excitation at room temperature. (a) Two-dimensional fluorescence decay map. (b) Decay traces at selected wavelengths with fits. (c) Evolution-associated spectra obtained from global fitting.

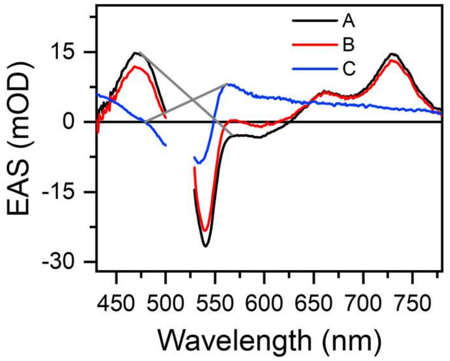

Figure S8. The triplet yield was estimated for 0.1% Am-NDI-doped in NDI single crystals by calculating the ratio of the GSB amplitudes of the longest (C) and shortest (A) lived kinetic components in the EAS. The procedure is detailed in ref.[2]

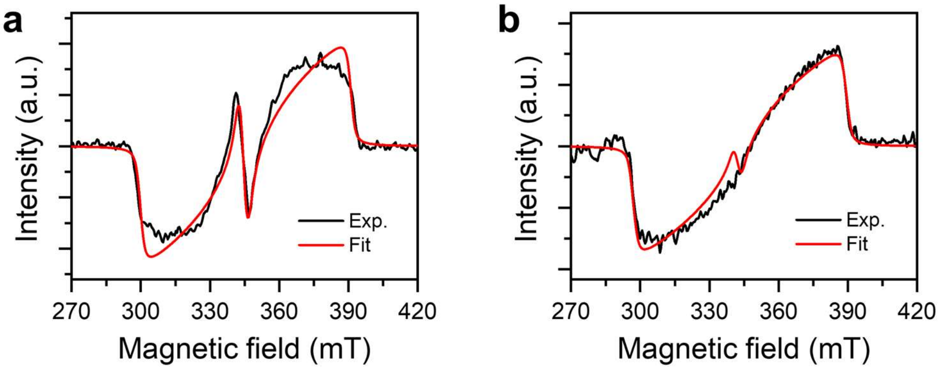

Figure S9. Time-resolved EPR spectra of (a) Am-NDI in toluene and (b) 0.1% Am-NDI-doped NDI crystal powders, averaged over 150–300 ns at 85 K. Spectra were simulated using the following parameters: D = 1320 ± 20 MHz, E = -416 ± 6 MHz. For Am-NDI in toluene, polarization parameters were P_x = 0.62, P_y = 0, P_z = 0.38; for the doped crystal, P_x = 0.9, P_y = 0, P_z = 0.1.

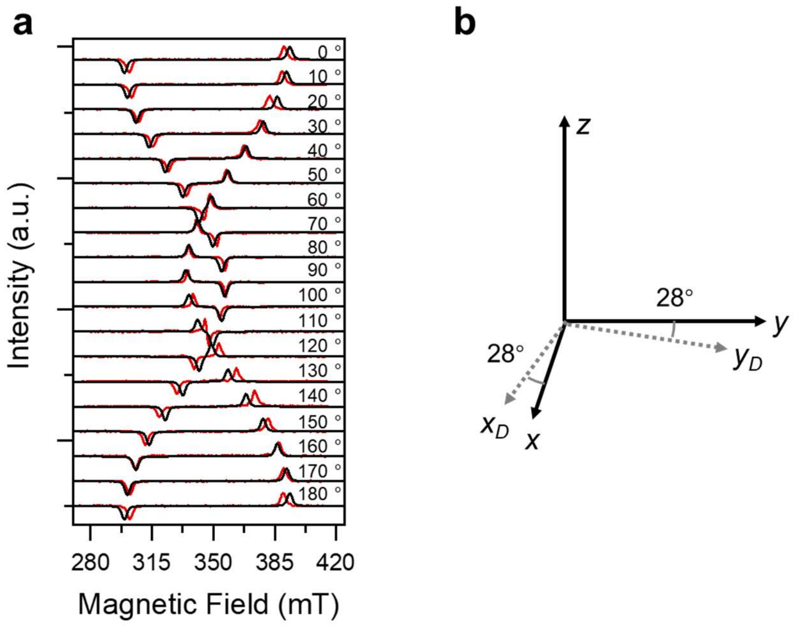

Figure S10. (a) Simulated angle-dependent TREPR spectra (black) overlaid with the experimental spectra (red) of the Am-NDI-doped NDI single crystal. The experimental data show no clear anisotropy in the linewidth, likely due to dominant inhomogeneous broadening. (b) Orientation relationship between the molecular frame (xyz) and the ZFS tensor frame (xDyDz) used in the simulations. The z-axes of the two frames are collinear, while the xy-plane of the ZFS tensor is rotated by ~28° relative to the molecular frame.

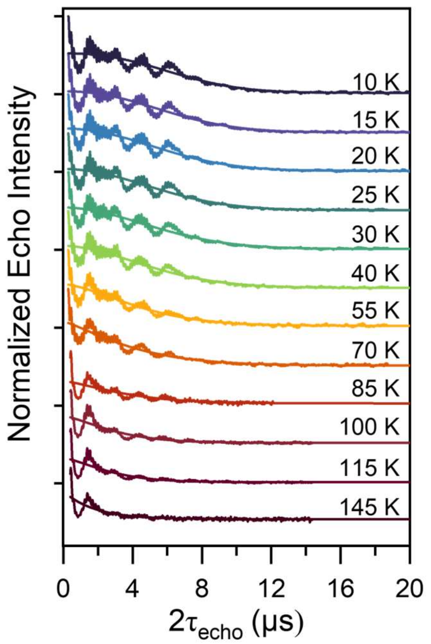

Figure S11. Representative temperature-dependent coherence decay traces of a 0.1% Am-NDI doped in an NDI single crystal collected using the Hahn echo pulse sequence (τ_{DAF} = 1.4 μs). The crystal was oriented at θ = 0°.

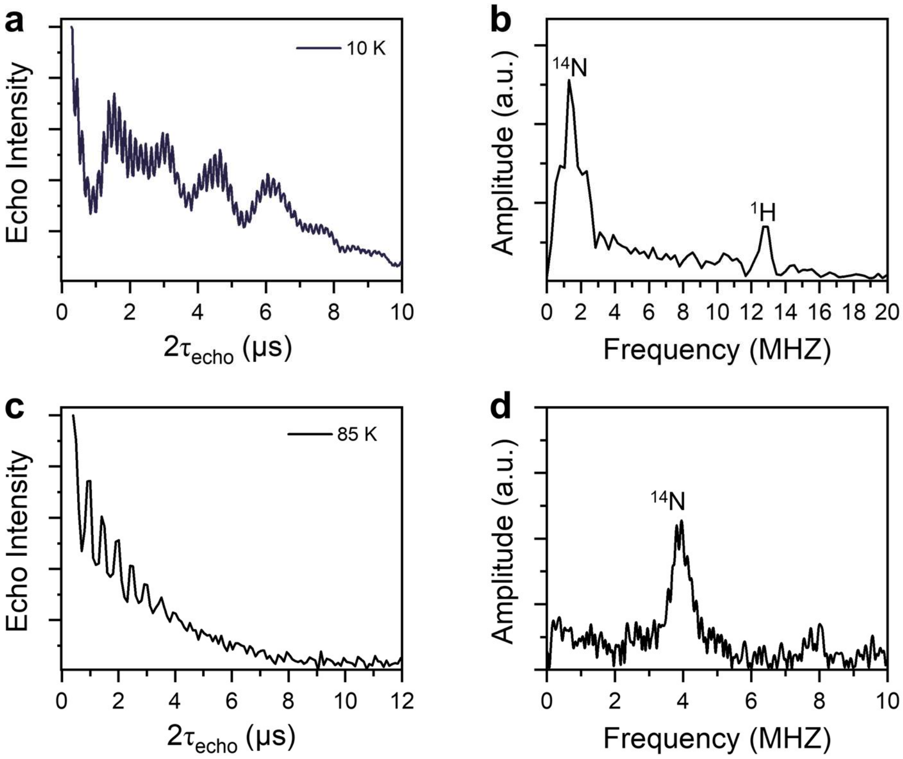

Figure S12. (a) Spin coherence decay trace with oscillations of a 0.1% Am-NDI doped in an NDI single crystal at 10 K, collected using X-band and a Hahn echo sequence. (b) Corresponding Fourier transform (FFT) of the data shown in (A). (c) Spin coherence decay trace with oscillations at 85 K, collected using Q-band and a Hahn echo sequence. (d) Corresponding FFT of the data in (c). The peaks labeled "^{14}N" "^{1}H" in (b) and (d) correspond to modulations arising from hyperfine coupling with nitrogen and proton nuclei. Note that different single crystals were used for the two measurements.

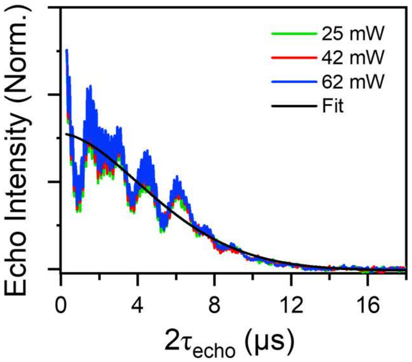

Figure S13. Spin coherence decay traces measured under varying laser excitation powers (τ_{DAF} = 1.4 μs).

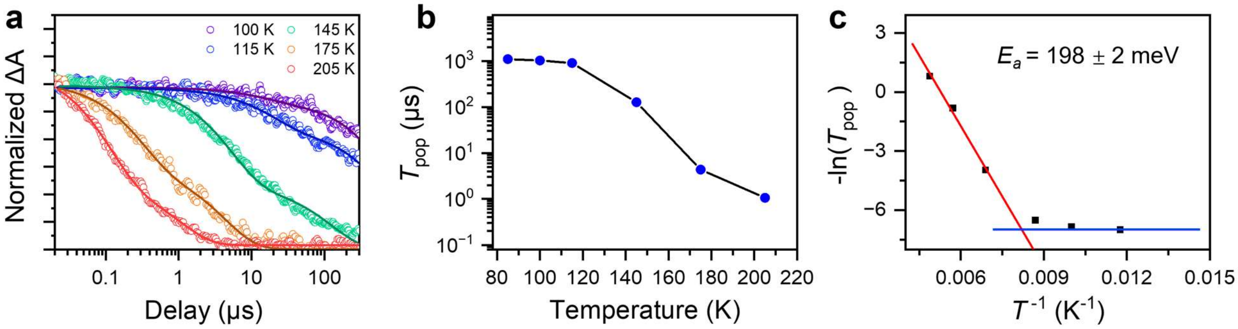

Figure S14. (a)Temperature-dependent transient absorption dynamics of a 0.1% Am-NDI doped in an NDI single crystal at 450 nm, normalized at 20 ns after ISC to reflect the triplet absorption of Am-NDI. The decay traces were fitted using a biexponential model, which may reflect heterogeneous triplet populations possibly associated with crystal defects. (b) Amplitude-weighted average triplet-state lifetimes extracted from the biexponential fits in (a), plotted as a function of temperature. (c) Arrhenius analysis of the temperature-dependent triplet-state lifetimes shown in (b), yielding an activation energy (E_a) of 198 ± 2 meV.

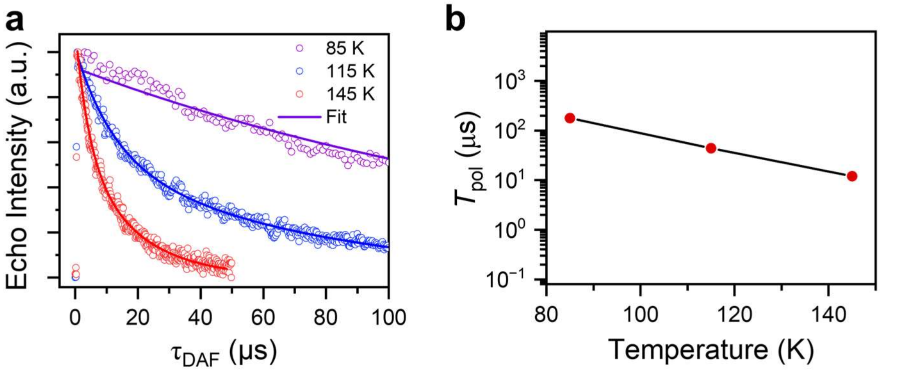

Figure S15. (a) Echo intensities as a function of τ_{DAF} at varying temperatures, along with mono-exponential fitting curves. (b) Temperature dependence of the polarization lifetimes of triplet states obtained by fitting the data shown in (A).

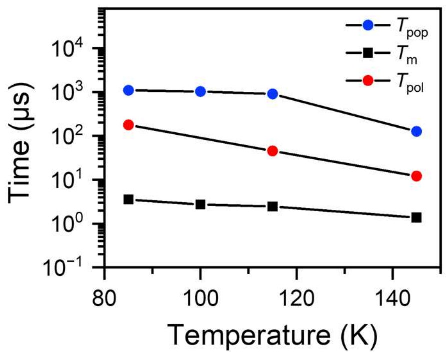

Figure S16. Comparison of three characteristic lifetimes of 0.1% Am-NDI-doped NDI single crystals: triplet population lifetime (blue), polarization lifetime (red), and spin coherence time T_m (black), measured at various temperatures.

7. References

1. Ke, H.; Jiao, C.; Qian, Y. H.; Lin, M. J.; Chen, J. Z., Naphthalene diimide templated synthesis of pillar [6] arenes. *Chin. J. Chem.* **2015,** *33*, 339-342.

2. Williams, M. L.; Schlesinger, I.; Jacobberger, R. M.; Wasielewski, M. R., Mechanism of ultrafast triplet exciton formation in single cocrystals of π-stacked electron donors and acceptors. *J. Am. Chem. Soc.* **2022,** *144*, 18607-18618.

3. Young, R. M.; Dyar, S. M.; Barnes, J. C.; Juricek, M.; Stoddart, J. F.; Co, D. T.; Wasielewski, M. R., Ultrafast conformational dynamics of electron transfer in exbox^{4+}⊂perylene. *J. Phys. Chem. A* **2013,** *117*, 12438-12448.

4. Palmer, J. R.; Williams, M. L.; Young, R. M.; Peinkofer, K. R.; Phelan, B. T.; Krzyaniak, M. D.; Wasielewski, M. R., Oriented triplet excitons as long-lived electron spin qutrits in a molecular donor–acceptor single cocrystal. *J. Am. Chem. Soc.* **2024,** *146*, 1089-1099.
